# A Study on the Function of Arginine in the Growth, Immunity, Antioxidant Activity, and Oxygen Carrying-Capacity of Juvenile Gibel Carp (*Carassius auratus gibelio*)

**DOI:** 10.3390/biotech13040056

**Published:** 2024-12-19

**Authors:** Yuqun Li, Lu Zhang, Mingchun Ren, Hualiang Liang, Haifeng Mi, Dongyu Huang

**Affiliations:** 1 Wuxi Fisheries College, Nanjing Agricultural University, Wuxi 214081, China; 2 Tongwei Agricultural Development Co., Ltd., Key Laboratory of Nutrition and Healthy Culture of Aquatic Livestock and Poultry, Ministry of Agriculture and Rural Affairs, Healthy Aquaculture Key Laboratory of Sichuan Province, Chengdu 610093, China; 3 Key Laboratory of Integrated Rice-Fish Farming Ecology, Ministry of Agriculture and Rural Affairs, Fresh-Water Fisheries Research Center, Chinese Academy of Fishery Sciences, Wuxi 214081, China

**Keywords:** juvenile Gibel carp, growth performance, antioxidant capacity, immune response, oxygen carrying capacity

## Abstract

An eight-week trial was designed to study the effects of arginine (Arg) supplemented diets on the growth, immunity, antioxidant activity, and oxygen-carrying capacity of juvenile Gibel carp (*Carassius auratus gibelio*). A total of 300 fish (27.53 ± 0.03 g) were randomized into 15 equal groups and fed on diets with graded Arg levels: 0 (control), 0.2%, 0.4%, 0.6%, and 0.8% (*w*/*w*). The results showed that final body weight (FBW), weight gain rate (WGR), and specific growth rate (SGR) all increased and then declined with increasing levels of Arg supplementation, while feed conversion ratio (FCR) showed the opposite trend. In addition, the fish’s whole-body crude protein and ash content had no remarkable difference at different levels of Arg addition (*p* > 0.05). Supplementation with 0.6% and 0.8% Arg significantly increased plasma alanine transaminase (ALT) activity (*p* < 0.05). The malondialdehyde (MDA) levels and superoxide dismutase (SOD) activities of the liver were not significantly different between the different levels of Arg supplementation (*p* > 0.05), while catalase (CAT) activity was significantly increased with 0.4% Arg supplementation levels (*p* < 0.05). The 0.8% Arg supplementation greatly increased the expression of hepatic-related genes to the Nrf2 signaling pathway, including *sod* and *gpx* (*p* < 0.05). However, the 0.8% Arg supplementation did not significantly increase the relative expression of genes related to the NF-κB signaling pathway, including *il-1β*, *il-8*, and *tnf-α* (*p* > 0.05). Similarly, the relative expression of hif-1 signaling pathway-related genes at 0.8% Arg supplementation was significantly elevated, including *hif-1α*, *epo*, and *vegf* (*p* < 0.05). Hence, Arg supplementation could promote growth and improve immune, antioxidant, and oxygen-carrying capacity in juvenile Gibel carp.

## 1. Introduction

Fish are an essential component of people’s daily diets, providing high-quality protein and unsaturated fatty acids [1]. Therefore, aquaculture is an important way to ensure humanity’s protein supply. However, with the fast development of high-density and intensive aquaculture models, aquaculture is facing many challenges. The likelihood of decreased immunity and hypoxia affecting live aquatic organisms has increased significantly [2]. Scientists have attempted to reverse this situation through the extensive use of antibiotics and other medicines [3,4]. However, many of these medicines are no longer suitable for aquaculture owing to increasing environmental pressures and restrictions on antibiotic use. Thus, nutritional modification is an important way to boost fish immune systems and reduce the risk of disease [5].

Amino acids are essential nutrients that regulate disease and support physiological functions. In recent years, the nutritional physiological role of amino acids has been increasingly correlated to growth [6,7]. Arginine (Arg) is an essential amino acid in all fish [8] and is the most versatile amino acid. Arg is involved not only in protein synthesis but also in a number of metabolic pathways, such as the production of urea, the metabolism of glutamate and proline, and the synthesis of creatine and polyamines [9,10]. Previous studies have shown that dietary Arg supplementation can positively affect fish growth [11,12,13]. Adding 1% Arg to feed significantly enhanced the final body weight (FBW), specific growth rate (SGR), feed conversion rate (FCR), and survival rate (SR) of hybrid striped bass (*Morone saxatilis*) [14]. A fish’s ability to digest and absorb nutrients in the intestine also affects its growth performance [15]. Arg significantly increased the depth of gut folds and the height of villi in hybrid striped bass [14]. In addition, Arg can be used as a dietary supplement: Atlantic salmon (*Salmo salar*) fed Arg-supplemented diets have shown a trend toward higher specific feed rate (SFR) and specific growth rate (SGR) and increased final body weight [16].

Moreover, Arg plays a vital role in mediating the immune response [17,18] and antioxidant capacity [19,20] of fish. Appropriate Arg levels stimulate non-specific immunity and improve disease resistance and fish survival [21]. As the only substrate for NO synthesis, Arg could regulate the NF-κB pathway by mediating toll-like receptors (TLRs) and then inhibit the expression of inflammatory response-related genes, such as tumor necrosis factor-α (*tnf-α*) and interleukin-1β (*il-1β*) [22,23,24]. Furthermore, Arg is essential for the antioxidant capacity of fish; it can activate the Nrf2 signaling pathway, upregulating the expression of nrf2 and reducing oxidative stress [25]. Activation of this pathway upregulates antioxidant genes such as superoxide dismutase (*sod*), catalase (*cat*), and glutathione peroxidase (*gpx*) [26] and enhances the antioxidant capacity of Jian carp (*Cyprinus carpio var.Jian*) [27], grass carp (*Ctenopharyngodon idella*) [25], and blunt snout bream (*Megalobrama amblycephala*) [28]. Furthermore, HIF-1 (hypoxia-inducible factor), a transcription factor regulated by oxygen levels in vivo, plays an active role in oxygen homeostasis in fish. Numerous genes downstream of HIF-1 have been found to enhance oxygen transport, including those that affect erythropoiesis, iron metabolism, angiogenesis, vascular tone, and oxygen consumption [29,30,31]. Increased *hif-1α* downstream gene expression facilitates adaptation to hypoxia and improves hypoxia tolerance in the doughnut bream [32]. As a precursor of nitric oxide, Arg enhances oxygen transport, and in recent years, it has been found to play an important role in regulating the oxygen-carrying capacity of animals. However, there are few related studies, especially on fish.

As a significant aquaculture species, Gibel carp (*Carassius auratus gibelio*) is found throughout Asia and Europe. People in China and other countries enjoy eating carp as a delicacy. However, with changes in the culture environment, Gibel carp is highly susceptible to stress and other effects in culture. Therefore, finding the right supplements to add to animal feed could improve animal health; in addition, there have been a few studies on feed supplementation for Gibel carp [33,34,35]. However, Arg is a beneficial feed supplementation to enhance fish growth and immune response, and research on the use of Arg as a feed supplementation for the health and welfare of Gibel carp is limited. Thus, this study will attempt to determine if Arg supplementation can improve growth, immune responses, antioxidative stress, and oxygen-carrying capacity in this species.

## 2. Materials and Methods

### 2.1. Diets

The base level of Arg in the feed is 2.19%, which meets the Arg requirement of juvenile Gibel carp at 2.0% [36]. On this basis, we designed five Arg levels: 0 (control), 0.2%, 0.4%, 0.6%, and 0.8% (*w*/*w*), which were replaced by equal proportions of glycine to ensure consistent feed protein. The fundamental dietary composition is described in Table 1. All components were thoroughly ground and passed through a 180 μm mesh sieve, and the diets were fixed using trial recipes. The feed was well mixed with oil and water. The mixed components were processed into 1 mm expanded granules using a machine for puffing aquatic feed (Jiangsu Muyang Holdings Co., Ltd., Yangzhou, China). Before use, the feed was dried, placed in a zip-lock bag, and maintained at 4 °C.

### 2.2. Feeding Procedure

Before the formal trials, the fish were fed general meals (Tong-Wei, Wuxi, China) for two weeks to acclimate them to their surroundings. Three hundred healthy juvenile Gibel carp with an initial weight of 27.53 (±0.03 g) were randomly distributed into 15 nets (1 m × 1 m × 1 m) with 20 fish in each cage stochastically. The eight-week feeding trial used a satiety feeding strategy (stop coming up to feed). Gibel carp were given two equal meals twice daily at 7 a.m. and 5:30 p.m. The water’s temperature and pH were measured daily throughout the trial: the range of water temperature was 28–32 °C, the pH was 7.0–7.8, the dissolved oxygen concentration remained at 6.0–7.8 mg/L, and the ammonia nitrogen content was less than 0.1 mg/L.

### 2.3. Sampling and Preservation

Before sampling, the fish were starved for twenty-four hours. To determine growth indicators, we then recorded the number and weight of the fish in each cage. Five fish were taken from each cage, three fish were taken out to gather liver and blood samples, and two were analyzed for general composition. The fish specimens were rendered unconscious using MS-222 (100 mg/L) before obtaining liver and blood samples. After drawing blood from the tail vein, the upper plasma was separated by centrifuging the mixture for ten minutes at 4000 rpm. As soon as blood was drawn, the fish was dissected to obtain liver samples. Liver and serum samples were kept at −80 °C for storage.

### 2.4. Analytical Methods

Whole-body fish and feed were analyzed for protein, moisture, crude lipid, and ash content according to the AOAC (2003) method and our previous study [37]; crude protein (N × 6.25) was determined with the Kjeldahl method after acid digestion; dry matter was determined after drying in an oven at 105 °C until constant weight; ash content was determined by incineration in a muffle furnace at 560 °C for 5 h; and lipid content was determined by ether extraction using Soxhlet. The biochemical levels of the blood samples were measured using a Mindray BS-400 autoanalyzer (Shenzhen, China). The antioxidant indices were measured as previously described [38]. Table 2 shows the detailed procedures, reagent kits, and devices used.

### 2.5. RNA Extraction and Real-Time PCR Analysis

Hepatic RNA was extracted using RNAiso Plus reagent (Vazyme, Nanjing, China), with an acceptable A260/280 ratio between 1.8 and 2.0 for further experiments. Real-time PCR analyzed the Ct value for different genes with a One Step SYBR Prime Script TM PLUS RT-PCR kit (Takara, Dalian, China) using CFX96 Touch (Bio-Rad, Singapore). Be-ta-actin (*β-actin*) was used as the internal reference gene owing to its steady and high expression levels, and no discernible variation was seen. Table 3 shows the gene sequences. Sangon Biotech (Shanghai) Co., Ltd. synthesized all primers. Some of these were based on references from earlier research, and the remainder were created online using Primer Premier 6.0. Finally, the gene expression results were analyzed using the standard curve approach [39].

### 2.6. Statistical Analysis

The data were subjected to normality and homogeneity tests where necessary, and then data were analyzed via one-way analysis of variance (ANOVA) using SPSS (20.0) and compared for significance using Tukey’s test. The data were recorded as mean ± standard error. Significant differences between values are represented with different alphabetical superscripts (*p* < 0.05).

## 3. Results

### 3.1. Growth Performance and Whole-Body Composition

**Figure 1:**
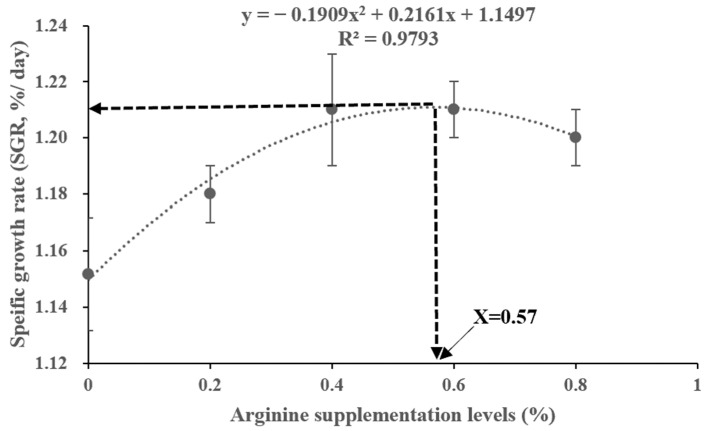
Quadratic regression model analysis of specific growth rate (SGR) against graded different supplementation levels of Arg.

**Figure 2:**
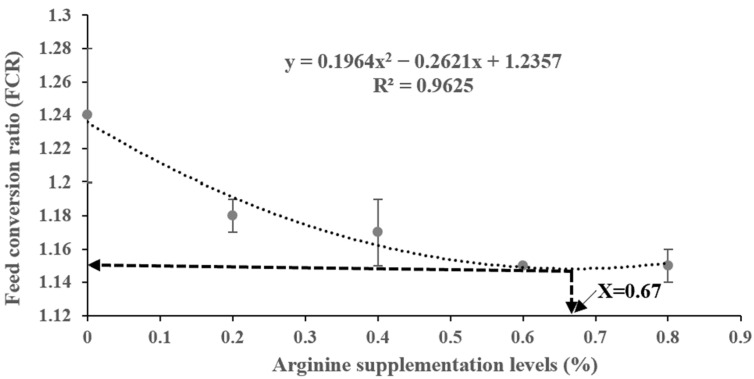
Quadratic regression model analysis of feed conversion ratio (FCR) against graded different supplementation levels of Arg.

Table 4 shows the growth performance results. The FBW and WGR values all showed an increasing trend up to the 0.4% Arg supplementation group, followed by a declining trend; the effects of the Arg supplementation levels were not statistically different (*p* > 0.05). The SGR was markedly higher in the 0.4% and 0.6% Arg supplementation groups than in the 0% (control) group (*p* < 0.05), and the FCR was significantly lower in the 0.6% and 0.8% Arg supplementation groups than in the 0% (control) group (*p* < 0.05). We compared the R^2^ of the SGR and FCR quadratic, linear, and broken linear regression models (SGR: quadratic regression R^2^ = 0.9793 > linear regression R^2^ = 0.6499 > broken linear regression R^2^ = 0.487; FCR: quadratic regression R^2^ = 0.9625 > linear regression R^2^ = 0.8047 > broken linear regression R^2^ = 0.497). Hence, we chose quadratic regression models based on the highest value of R^2^; quadratic regression analyses yielded optimal Arg supplementation levels for juvenile Gibel carp for SGR and FCR, which were 0.57% and 0.67% in the dry diet, respectively (Figure 1 and Figure 2). Table 5 shows that the whole-body water, fat, ash, and protein contents were not significantly affected by the feed treatments in this study (*p* > 0.05).

### 3.2. Plasma Biochemical Parameters

Table 6 shows the plasma parameter results. The 0.6% and 0.8% Arg supplementation groups showed ALT levels that were significantly higher than the 0% (control) group (*p* < 0.05). ALB and AST levels did not markedly differ across the groups (*p* > 0.05). TC and TG levels did not differ significantly between groups (*p* > 0.05).

### 3.3. Liver Antioxidant Parameters

The liver antioxidant parameter results are presented in Table 7. The CAT activity increased to 0.4% and then decreased (*p* < 0.05). Compared with the 0% group, there are no significant differences in the SOD activity and MDA levels in Arg diets (*p* > 0.05).

### 3.4. Inflammatory Factor Gene Expression

**Figure 3:**
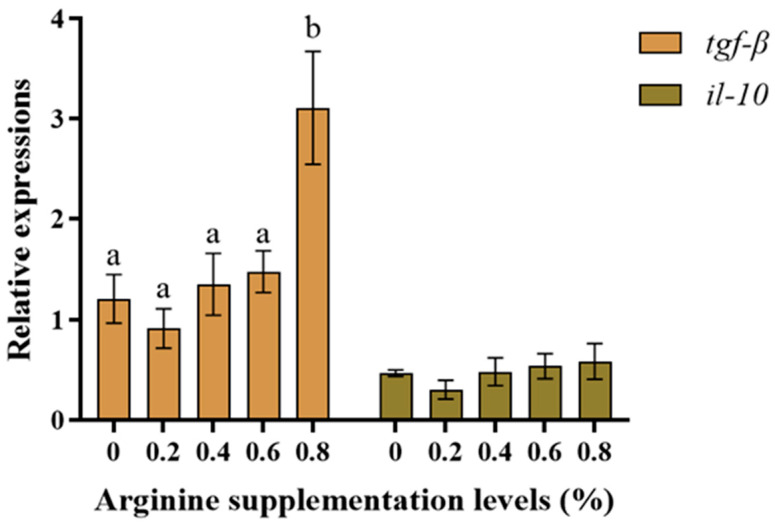
The anti-inflammatory gene expression in Gibel carp. Those significantly different from each other are represented by different letters (a and b).

**Figure 4:**
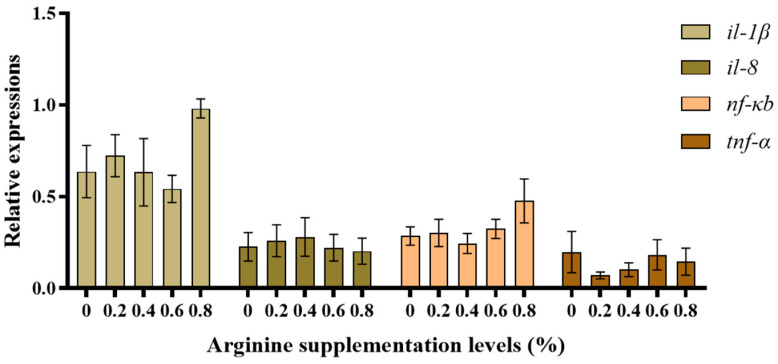
The pro-inflammatory gene expression in Gibel carp.

Figure 3 shows the anti-inflammatory gene expression results. The *tgf-β* mRNA expression was highest in the 0.8% Arg supplementation group and was significantly higher than in the 0% (control) group (*p* < 0.05). There was a tendency for *il-10* mRNA expression to increase; however, there was no significant difference compared to the control group (*p* > 0.05). Figure 4 shows the pro-inflammatory gene expression results. The *il-1β* and *nf-κb* mRNA expression showed no significant difference compared to the control group (*p* > 0.05). There was no statistical difference in *tnf-α* and *il-8* mRNA expression across the groups (*p* > 0.05).

### 3.5. Antioxidant-Related Gene Expression

**Figure 5:**
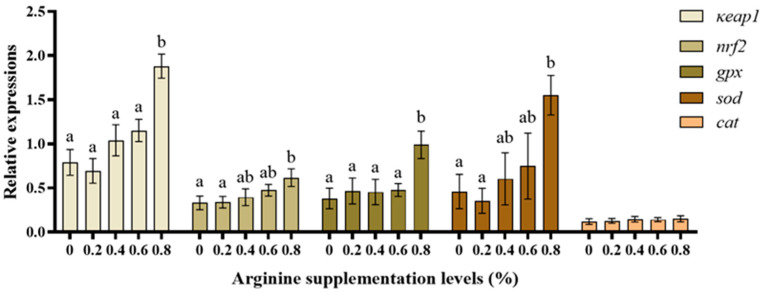
The antioxidant-related gene expression in Gibel carp. Those significantly different from each other are represented by different letters (a and b).

Figure 5 shows the antioxidant-related gene results. The *nrf2* mRNA expression was significantly higher in the 0.8% Arg supplementation group than in the 0% (control) group (*p* < 0.05). The *κeap1* mRNA expression was significantly higher in the 0.8% Arg supplementation group than in the 0% (control) group (*p* < 0.05). The sod and *gpx* mRNA expression was highest in the 0.8% Arg supplementation group (*p* < 0.05). There was no difference between the groups in *cat* mRNA expression, which showed the same trend (*p* > 0.05).

### 3.6. HIF-1 Pathway-Associated Gene Expression

**Figure 6:**
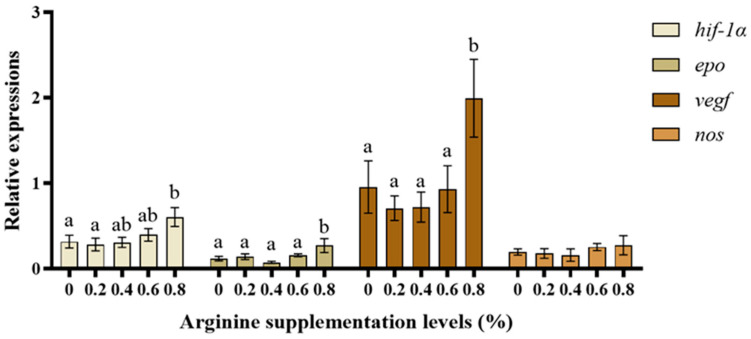
The HIF-1 pathway-related gene expression in Gibel carp. Those significantly different from each other are represented by different letters (a and b).

Figure 6 shows gene expression results related to the HIF-1 pathway. The *hif-1α*, *epo*, and *vegf* mRNA expression in the 0.8% Arg supplementation group were markedly higher than in the 0% (control) group (*p* < 0.05). There was no significant difference in *nos* mRNA expression between the groups (*p* > 0.05).

## 4. Discussion

In this study, Arg-supplemented diets improved the growth performance and feed efficiency of juvenile Gibel carp. Although no significant changes were observed in FBW and WGR after Arg supplementation, 0.4–0.6% Arg supplementation significantly increased SGR compared with that of the control group, and 0.6–0.8% Arg supplementation significantly decreased the FCR compared with that of the control group. The increase in SGR and FCR may reveal this theory that Arg in the feed has a beneficial effect on the growth of juvenile Gibel carp. There are quite a few literature studies on Gibel carp with feed supplementation [33,34,35]; while Arg is a beneficial feed supplementation, there was no research on Gibel carp. Arg is an essential amino acid for fish; therefore, any excess or shortage in the diet may reduce feed utilization and growth performance [44]. In a previous study, the FBW of rainbow trout (*Oncorhynchus mykiss*) was improved when Arg feed levels increased from 1.72% to 3.09% [45]. The WGR and feed efficiency of hybrid striped bass were also improved when feed was supplemented with 1.45% to 1.55% Arg levels [46]. In addition, studies have shown that Arg supplementation above the prescribed minimum requirement can improve feeding and growth in Atlantic salmon [16]. Feed supplemented with the dipeptide or free form of Arg significantly affected the FBW, WGR, and SGR of juvenile South American pacu (*mesopotamicus Piaractus mesopotamicus*) [12]. Furthermore, the growth-promoting effect of Arg has been confirmed in several other fish species, including largemouth bass (*Micropterus salmoides*) [47], spotted catfish (*Ictalurus punctatus*) [48], and Nile tilapia (*Oreochromis niloticus*) [49]. The results of our experiments support the current data for juvenile Gibel carp, suggesting that Arg supplementation performs a critical function in regulating the growth performance of fish. This is because Arg is an essential amino acid for fish and participates in protein synthesis, promoting growth [50].

Arg supplementation in the diet increased the crude protein content of juvenile Gibel carp to an extent; however, there was no discernible difference between the whole-body crude protein, crude fat, and ash levels in the supplemented groups and those of the control group. These results agree with those for juvenile golden pompan (*Trachinotus ovatus*) [51] and juvenile Nile tilapia (*Oreochromis niloticus*) [52]. However, one study found that adding 9–12 g/kg of Arg to the feed significantly increased the overall protein content and reduced lipid deposition in juvenile Asian red-tailed catfish (*Hemibagrus wyckoiides*) [53]. Thus, the effect of feed supplementation with amino acids on whole-body composition may be related to the species of fish. As a vital metabolic transport system, one of the prominent functions of blood is to regulate the lipid metabolism in the organism and blood lipid levels can be used as an indicator of lipid metabolism [54]. TG and TC are lipid metabolism intermediates and are thus considered good indicators of lipolytic metabolism [55]. In this study, TG and TC contents did not significantly differ between the groups, while lower TC and TG contents were recorded in the 0.6% and 0.8% groups, similar to the results of dietary supplementation with leucine in Gibel carp [56]. ALT reached its maximum in the 0.8% Arg supplementation group, and AST did not differ between groups, possibly because of vigorous metabolism in the body due to Arg supplementation rather than liver injury [57] because AST and ALT also play an important role in fish nutrient metabolism [58]. In this study, ALB did not show significant changes, similar to the results for juvenile Asian red-tailed catfish (*Hemibagrus wyckoiides*) [53].

Nrf2 is one of the main cellular oxidative stress regulators, and the *nrf2-κeap1* signaling pathway can modulate the expression of several detoxification enzymes and antioxidant-protein-related genes [59]. In this study, *nrf2* mRNA and *κeap1* mRNA expression levels were simultaneously upregulated in the 0.8% Arg supplementation group compared with the control group, indicating that the Nrf2 antioxidant system can only be activated when the supplementation level of Arg reaches a certain level. Normally, *κeap1* plays a significant negative regulatory role for *nrf2* and interacts with the Neh2de electronic domain of *nrf2* [60]. In addition, *gpx* and *sod* mRNA levels were highest in the 0.8% Arg supplementation group. Our results showed a dose-dependency rise in the *gpx* and *sod* gene expression, which indicates that high levels of Arg increased the antioxidant functions of Gibel carp, like male zebrafish (*Danio rerio*) [61]. The antioxidant defense systems of organisms also include SOD, CAT, GPX, and GSH [27]. In the present study, Arg feed supplementation increased CAT activity but did not have any significant effect on SOD activity in the liver. Since antioxidant enzymes are essential for fish to avoid oxidative stress and scavenge superoxide anion and hydroxyl radicals [62], exogenous Arg supplementation may increase their antioxidant capacity. Similar results were reported in hybrid grouper (*Epinephelus fuscoguttatus* ♀ *× Epinephelus lanceolatus* ♂) [63] and yellow catfish (*Pelteobagrus fulvidraco*) [64]. Various reports have shown that antioxidant enzyme activities are closely related to their gene expression. It was further shown that Arg improves and enhances antioxidant capacity. Therefore, adding Arg to the diet is beneficial for the antioxidant capacity of juvenile Gibel carp.

In the past few years, Arg-mediated immunomodulation has attracted particular interest [65,66,67]. Arg has multiple functions, including participation in protein synthesis, urea production, polyamine synthesis, proline and guanidinium synthesis, and the endocrine and reproductive regulatory processes. Therefore, its involvement in the immune response of fish may be crucial for managing their health throughout the feeding cycle. This is a key reason why Arg supplementation is considered a strategy for improving immune responses [68,69]. For example, in turbot, Arg supplementation improved the immunosuppressive response because of high stocking density [70]. In addition, studies on mirror carp (*Cyprinus carpio*) have shown that dietary Arg supplementation upregulates anti-inflammatory genes in the gut [62]. Our results showed *il-1β* expression in the liver was highest in the liver at 0.8% Arg level, in line with Chen et al. [71] and Hoseini et al. [72], who found upregulated *il-1β* and *tnf-α* levels in the heads and kidneys of carp after Arg supplementation. Under normal conditions, this effect on pro-inflammatory cytokines could be interpreted as enhancing the immune response, preparing fish to face pathogens. Supporting this hypothesis, Chen et al. proposed that Arg enhances the immune response and increases resistance to Aeromonas hydrophila in Jian carp by upregulating the expression of pro-inflammatory genes [71]. That study’s findings show that 0.8% Arg supplementation significantly upregulated the relative expression of *tgf-β*, suggesting that appropriate Arg levels may attenuate tissue inflammation and reconcile body homeostasis by increasing the gene expression of anti-inflammatory factors such as *tgf-β*. Taken together with our results, this might indicate that Arg supplementation improves growth and immune responses.

Hif-1α is an upstream gene of the hypoxia signaling pathway and can regulate the expression of various genes in this pathway, possibly modulating fish responses to hypoxic environments [72,73,74,75,76]. *Epo* and *vegf* are important factors that manage erythropoiesis and angiogenesis [77]. In this study, only the 0.8% Arg supplementation group exhibited significantly greater relative *hif-1α*, *vegf*, and *epo* gene expression than the control group in a normoxic environment, indicating that there was a dose-dependent effect of Arg supplementation on the organisms, which is related to the production and degradation of vessels and hemoglobin [63,64,65,66,67,68,69,70,71,72,73,74,75]. Fish can adapt to prolonged exposure to hypoxia by increasing blood volume with hemoglobin affinity [78], indicating that Arg supplementation might improve their hypoxia tolerance and the ability to transport oxygen. Similarly, the oxygen consumption of juvenile European sea bass (*Dicentrarchus labrax*) during feed deprivation is similar under normoxic and hypoxic conditions, suggesting that fish can adapt to prolonged exposure to hypoxia by increasing blood volume through hemoglobin affinity [79]. Hemoglobin proteins are oxygen sensors in cells [80,81], and HIF-1 can receive the peroxides and reactive oxygen species signaling molecules produced by these sensors [82]. In summary, Arg supplementation enhances the oxygen-binding capacity of hemoglobin, which is why it improves feed utilization.

## 5. Conclusions

In this study, binding key immune factor expression levels and growth performance, adding exogenous Arg supplementation at 0.4–0.6% positively affected the growth, antioxidant capacity, and immune capacity of juvenile Gibel carp, with some dose-dependency. Arg also plays an important role in enhancing the oxygen-binding capacity of their hemoglobin. This information will provide a basis for developing Arg functional additives.

## Figures and Tables

**Table 1:** Formulation and proximate composition of the experimental feed (% dry matter).

Ingredients	Diet 1	Diet 2	Diet 3	Diet 4	Diet 5
Fish meal	14	14	14	14	14
Chicken meal	4	4	4	4	4
Soybean meal	22	22	22	22	22
Cotton meal	5	5	5	5	5
Rapeseed meal	22	22	22	22	22
Wheat meal	14.15	14.15	14.15	14.15	14.15
Rice bran	10	10	10	10	10
Soybean oil	4	4	4	4	4
Monocalcium phosphate	2	2	2	2	2
F11V Vitamin Premix for Carnivorous Fish	0.2	0.2	0.2	0.2	0.2
F29M Trace element premix for omnivorous fish	2	2	2	2	2
L-lysine (98.5%)	0.3	0.3	0.3	0.3	0.3
L-methionine	0.1	0.1	0.1	0.1	0.1
Vc Phospholipid	0.05	0.05	0.05	0.05	0.05
Choline Chloride (60%)	0.2	0.2	0.2	0.2	0.2
L-Glycine	0.8	0.6	0.4	0.2	0
L-Arg	0	0.2	0.4	0.6	0.8
Analyzed proximate composition					
Crude protein (%)	38.45	38.71	38.78	38.78	38.46
Crude lipid (%)	6.25	6.18	6.62	6.46	6.22
Gross energy (MJ/Kg)	15.81	15.76	15.83	15.80	15.77

Note: The protein content of the fish meal, soybean meal, rapeseed meal, chicken meal, and cotton meal was 65.6%, 46.0%, 39.2%, 62.5%, and 53.7%, respectively, and the crude lipid content of the fish meal, soybean meal, rapeseed meal, chicken meal, and cotton meal was 9.5%, 4.3%, 6.1%, 10%, and 1.4%, respectively. These values are part of the composition and analysis for each ingredient. All the ingredients were sourced from Wuxi Tong-Wei Feedstuffs Co., Ltd., Wuxi, China.

**Table 2:** Primary methodology and analytical equipment.

Items	Methods	Assay Kits/Testing Equipment
Composition of diets/ingredients/Fish		
Moisture	Drying method	Electric blast drying oven (Shanghai Yiheng Scientific Instrument Co., Ltd., Shanghai, China)
Protein	Kjeldahl	Auto kieldahl apparatus: Hanon K1100 (Jinan Hanon Instruments Co., Ltd., Jinan, China)
Lipid	Soxhlet	Auto fat analysis: Hanon SOX606 (Jinan Hanon Instruments Co., Ltd., Jinan, China)
Ash	Combustion	Muffle: XL-2A (Hangzhou Zhuochi Instrument Co., Ltd., Hangzhou, China)
Plasma parameters		
Albumin (ALB) Total cholesterol (TC) Triglyceride (TG)	International Federation of Clinical Chemistry recommended	Assay kits purchased from Mindray Medical International Ltd. (Shenzhen, China); Mindray BS-400 automatic biochemical analyzer (Mindray Medical International Ltd., Shenzhen, China).
Alanine transaminase (ALT) Aspartic transaminase (AST)		
Superoxide dismutase (SOD) Malondialdehyde (MDA) Catalase (CAT)	Liver antioxidant capacity WST-1 method TBA method Ammonium molybdenum acid method	Assay kits purchased from Jian Cheng Bioengineering Institute (Nanjing, China), Spectrophotometer (Thermo Fisher Multiskan GO, Shanghai, China).

**Table 3:** Real-time PCR primer sequences used in the present study.

Genes	Forward Primer (5′-3′)	Reverse Primer (5′-3′)	Accession Number/Reference
*il-10*	AGTGAGACTGAAGGAGCTCCG	TGGCAGAATGGTGTCCAAGTA	[40]
*tgf-β*	GTTGGCGTAATAACCAGAAGG	AACAGAACAAGTTTGTACCGATAAG	[41]
*il-1β*	GCGCTGCTCAACTTCATCTTG	GTGACACATTAAGCGGCTTCA C	[41]
*il-8*	ATTGGTGAAGGAATGAGTCT	CCACAGATGACCTTGACAT	KC184490.1
*tnf-α*	CATTCCTACGGATGGCATTTACTT	CCTCAGGAATGTCAGTCTTGCAT	[41]
*nf-κb*	GCTCTGACTGCGGTCTTATAC	GCGCTTCATCGAGGATAGTT	[42]
*gpx*	GAAGTGAACGGTGTGAACGC	GATCCCCCATCAAGGACACG	DQ983598.1
*cat*	TGAAGTTCTACACCGATGAG	CTGAGAGTGGACGAAGGA	XM_026238665.1
*sod*	TCGGAGACCTTGGTAATGT	CGCCTTCTCATGGATCAC	JQ776518.1
*κeap1*	CTCCGCTGAATGCTACAA	GGTCATAACACTCCACACT	XM_026245355.1
*Nrf2*	TACCAAAGACAAGCAGAAGAAACG	GCCTCGTTGAGCTGGTGTTTGG	[43]
*hif-1α*	CTGCCGATCAGTCTGTCTCC	TTTGTGGAGTCTGGACCACG	DQ306727.1
*vegf*	ATCGAGCACACGTACATCCC	CCTTTGGCCTGCATTCACAC	NM_131408.3
*epo*	CGAAGTGTCAGCATACCGGA	GCAGATGACGCACTTTTCCC	KC460317.1
*β-actin*	TCCATTGTTGGACGACCCAG	TGGGCCTCATCTCCCACATA	LC382464.1

Note: *tgf-β*, transforming growth factor-β; *il-10*, interleukin-10; *il-8*, interleukin-8; *il-1β*, interleukin-1β; *tnf-α*, tumor necrosis factor-α; *nf-κb*, nuclear factor kappa-β; *gpx*, glutathione peroxidase; *cat*, catalase; *sod*, superoxide dismutase; *nrf2*, nuclear factor erythroid 2-related factor 2; *κeap1*, recombinant kelch like ech associated protein 1; *hif-1α*, hypoxia-inducible factor 1-α; *vegf*, vascular endothelial growth factor; *epo*, erythropoietin; *β-actin*, beta-actin.

**Table 4:** Effect of Arg supplementation levels on growth performance.

Arg Addition Levels (%)	^1^ IBW(g)	^2^ FBW(g)	^3^ WGR (%)	^4^ SGR(%/day)	^5^ FCR
0	27.45 ± 0.08	80.13 ± 1.56	191.93 ± 0.06	1.15 ± 0.02 ^a^	1.24 ± 0.04 ^b^
0.2	27.52 ± 0.06	82.83 ± 0.44	201.00 ± 0.02	1.18 ± 0.01 ^ab^	1.18 ± 0.01 ^ab^
0.4	27.53 ± 0.03	84.81 ± 1.41	208.03 ± 0.05	1.21 ± 0.02 ^b^	1.17 ± 0.02 ^ab^
0.6	27.50 ± 0.06	84.50 ± 0.47	207.27 ± 0.02	1.21 ± 0.01 ^b^	1.15 ± 0.00 ^a^
0.8	27.47 ± 0.06	84.05 ± 1.07	206.00 ± 0.03	1.20 ± 0.01 ab	1.15 ± 0.01 a
*p*-value					
Linear trend	0.926	0.022	0.019	0.025	0.008
Quadratic trend	0.487	0.010	0.011	0.017	0.012

Note: ^1^ Initial body weight (IBM). ^2^ Final body weight (FBM). ^3^ Weight gain rate (WGR) (%) = 100 × (FBW (g) *−* IBW (g))/IBW (g). ^4^ Specific growth rate (SGR) (%/d) = 100 × [(Ln (FBW (g)) *−* Ln (IBW (g)))/days]. ^5^ Feed conversion ratio (FCR) = dry feed fed (g)/wet weight gain (g). The mean ± standard error was used to characterize the data. Superscripts of different letters (a and b) indicate significant differences between groups (*p* < 0.05).

**Table 5:** Effect of Arg supplementation levels on whole body composition.

Arg Addition Levels (%)	Moisture (%)	Lipid (%)	Ash (%)	Protein (%)
0	73.86 ± 0.45	3.59 ± 0.32	5.42 ± 0.33	15.78 ± 0.53
0.2	73.70 ± 0.85	3.73 ± 0.82	5.67 ± 0.15	16.01 ± 0.11
0.4	74.54 ± 0.77	3.74 ± 0.68	4.68 ± 0.36	15.68 ± 0.3
0.6	73.01 ± 0.53	3.79 ± 0.42	5.07 ± 0.54	16.29 ± 0.22
0.8	75.52 ± 0.15	2.29 ± 0.37	5.15 ± 0.18	16.25 ± 0.26
*p*-value				
Linear trend	0.263	0.177	0.399	0.220
Quadratic trend	0.299	0.123	0.484	0.456

Note: The mean ± standard error was used to characterize the data.

**Table 6:** Effect of Arg supplementation levels on plasma parameters.

Arg Addition Levels (%)	ALB (g/L)	ALT (U/L)	AST (U/L)	TC (mmol/L)	TG (mmol/L)
0	8.59 ± 0.24	0.76 ± 0.1 ^ab^	166.3 ± 9.45	6.86 ± 0.58	1.33 ± 0.06
0.2	8.77 ± 0.42	0.44 ± 0.11 ^a^	169.44 ± 10.09	7.00 ± 0.35	1.29 ± 0.10
0.4	8.86 ± 0.41	1.12 ± 0.22 ^bc^	185.37 ± 13.85	7.10 ± 0.43	1.41 ± 0.13
0.6	8.19 ± 0.35	1.47 ± 0.18 ^c^	166.32 ± 9.78	6.21 ± 0.37	1.23 ± 0.09
0.8	7.96 ± 0.49	1.53 ± 0.23 ^c^	171.88 ± 9.49	6.20 ± 0.49	1.25 ± 0.10
*p*-value					
Linear trend	0.137	0.000	0.811	0.143	0.477
Quadratic trend	0.184	0.000	0.730	0.258	0.694

Note: The mean ± standard error was used to characterize the data. Superscripts of different letters (a, b, c) indicate statistically significant between groups (*p* < 0.05).

**Table 7:** Effect of Arg supplementation levels on liver antioxidant parameters.

Arg Addition Levels (%)	CAT (U/mgprot)	SOD (U/mgprot)	MDA (nmol/mgprot)
0	7.45 ± 2.37 ^a^	28.08 ± 0.65	6.00 ± 0.17
0.2	18.25 ± 2.09 ^bc^	26.90 ± 0.85	5.89 ± 0.64
0.4	23.15 ± 2.04 ^c^	26.81 ± 0.83	6.17 ± 0.66
0.6	18.42 ± 2.43 ^bc^	26.79 ± 0.64	5.99 ± 1.02
0.8	13.10 ± 1.75 ^ab^	26.25 ± 1.25	7.04 ± 1.02
*p*-value			
Linear trend	0.373	0.165	0.123
Quadratic trend	0.000	0.354	0.314

Note: The mean ± standard error was used to characterize the data. Superscripts of different letters (a, b, c) indicate significant differences between groups (*p* < 0.05).

## Data Availability

The original contributions presented in the study are included in the article, further inquiries can be directed to the corresponding authors.
